# Prioritizing test cases for deep learning-based video classifiers

**DOI:** 10.1007/s10664-024-10520-1

**Published:** 2024-07-22

**Authors:** Yinghua Li, Xueqi Dang, Lei Ma, Jacques Klein, Tegawendé F. Bissyandé

**Affiliations:** 1 SnT Centre, University of Luxembourg, Esch-sur-Alzette, Luxembourg; 2 The University of Tokyo, Tokyo, Japan

**Keywords:** Test input prioritization, Deep neural network, Learning to rank, Labeling

## Abstract

The widespread adoption of video-based applications across various fields highlights their importance in modern software systems. However, in comparison to images or text, labelling video test cases for the purpose of assessing system accuracy can lead to increased expenses due to their temporal structure and larger volume. Test prioritization has emerged as a promising approach to mitigate the labeling cost, which prioritizes potentially misclassified test inputs so that such inputs can be identified earlier with limited time and manual labeling efforts. However, applying existing prioritization techniques to video test cases faces certain limitations: they do not account for the unique temporal information present in video data. Unlike static image datasets that only contain spatial information, video inputs consist of multiple frames that capture the dynamic changes of objects over time. In this paper, we propose VRank, the first test prioritization approach designed specifically for video test inputs. The fundamental idea behind VRank is that video-type tests with a higher probability of being misclassified by the evaluated DNN classifier are considered more likely to reveal faults and will be prioritized higher. To this end, we train a ranking model with the aim of predicting the probability of a given test input being misclassified by a DNN classifier. This prediction relies on four types of generated features: temporal features (TF), video embedding features (EF), prediction features (PF), and uncertainty features (UF). We rank all test inputs in the target test set based on their misclassification probabilities. Videos with a higher likelihood of being misclassified will be prioritized higher. We conducted an empirical evaluation to assess the performance of VRank, involving 120 subjects with both natural and noisy datasets. The experimental results reveal VRank outperforms all compared test prioritization methods, with an average improvement of 5.76%$\sim$46.51% on natural datasets and 4.26%$\sim$53.56% on noisy datasets.

## Introduction

The rapid growth of multimedia on the Internet has led to an exponential increase in the number of videos being shared every minute. The popularity of short videos has further heightened the demand for video classification algorithms (Tran et al. 2018) to facilitate speedy user video recommendations (Covington et al. 2016). Specifically, video classification plays a crucial role in identifying and tracking objects in a variety of domains, such as accident detection (Ghosh et al. 2019; Agrawal et al. 2020; Bouhsissin et al. 2021). Given the crucial usage, the presence of bugs in video-oriented Deep Neural Networks (DNNs) can have severe real-world consequences, especially in safety-critical domains (Peng et al. 2021). Here, bugs refer to certain internal parameter weights within the video classification model that can lead to prediction errors when dealing with video inputs. For example, consider a highway scenario where the camera-equipped video classification model is specifically engineered to determine whether a given scene involves a car accident. In the event of an erroneous prediction, where the model misclassified the accident scene as a safe scenario, there is a risk of failing to issue a timely warning. This oversight can potentially result in severe consequences due to a lack of prompt assistance. Therefore, it is crucial to guarantee the quality of DNN models employed for video classification.

DNN testing (Sun et al. 2018) is widely recognized as an effective means of ensuring the quality of DNNs, including DNNs for video classification. However, a significant challenge in DNN testing lies in the high cost associated with labelling test inputs to verify the accuracy of DNN predictions. The general reasons include: 1) the test set is usually large-scale; 2) manual labelling is still mainstream; 3) labelling can require domain-specific expertise. Furthermore, in comparison to labeling image and text data, labeling video-type test inputs presents unique challenges, outlined as follows.

- Video data is characterized by its sequential composition of frames, establishing a temporal structure. Unlike static images or text, video data necessitates annotators to meticulously observe and analyze the content over time, frame by frame.
- Video datasets can contain multiple objects and events within a single frame, making it challenging to identify which objects or events should be labelled.
- Video datasets are typically much larger than image/text datasets, containing multiple frames per second, which can create a large volume of data to be labelled. This can be time-consuming and resource-intensive, requiring significant human labor.

To relieve the labelling cost problem, one effective way is test prioritization (Feng et al. 2020), which aims to prioritize bug-revealing test inputs (i.e., test inputs that are more likely to be misclassified by the DNN model) earlier in the testing process so that those test inputs can be labeled earlier. To this end, researchers have proposed several test input prioritization techniques to address the labelling-cost issue in DNNs (Wang et al. 2021; Feng et al. 2020). These techniques can be broadly categorized into coverage-based and confidence-based approaches. Coverage-based approaches, such as CTM (Yoo and Harman 2012), prioritize test inputs based on neuron coverage and adapt coverage-based prioritization techniques from traditional software testing (Yoo and Harman 2012; Lou et al. 2019). On the other hand, confidence-based approaches (Feng et al. 2020; Wang et al. 2021) assume that test inputs with lower model confidence are more likely to be misclassified and hence should be prioritized higher. DeepGini (Feng et al. 2020), a classical confidence-based test prioritization approach, considers a test input more likely to be misclassified if the model outputs similar prediction probabilities for each class. Wang et al. (2021) proposed PRIMA, which leverages mutation analysis and learning-to-rank methods to prioritize test inputs for DNNs.

However, when applying the aforementioned existing test prioritization methods to the scenario of video test inputs, certain limitations arise:

- The approaches mentioned above do not take into account the unique temporal information present in video data. In contrast to images and text, video inputs consist of multiple frames that capture the dynamic nature and temporal fluctuations of objects over time.
- The mutation-based test prioritization approach PRIMA is not applicable to video test inputs because the mutation rules of PRIMA are not adapted for video datasets.

In this paper, we propose VRank (**V**ideo Test Inputs **Rank**ing), the first test input prioritization technique tailored exclusively for video test inputs. The fundamental concept underlying VRank is that video-type tests with a higher probability of being misclassified by the evaluated DNN classifier are considered more likely to reveal faults and will be prioritized higher. To achieve this, we train a ranking model with the goal of predicting the probability of a given test input being misclassified by a DNN classifier. Specifically, the ranking model is trained using a dataset generated from the training sets of the evaluated DNN classifier. For a given video-type test, we generate four different types of features for the ranking model to make predictions: temporal features (TF), video embedding features (EF), prediction features (PF), and uncertainty features (UF). Ma et al. (2018b) previously demonstrated that test inputs located close to the decision boundary of the DNN classifier are more likely to be misclassified. Therefore, based on these four types of features, the ranking model can learn the test’s proximity to the DNN classifier’s decision boundary and, consequently, predict the probability of the test being misclassified by the model. We rank all test inputs in the target test set based on their misclassification probabilities. Videos with a higher likelihood of being misclassified are considered more likely to reveal faults. Consequently, these potentially misclassified videos will be prioritized higher. In the following, we provide detailed information about the four types of features generated for a specific test input.

- **Temporal Features(TF)** TF captures the unique temporal coherence inherent in a given video-type test. The primary objective of generating TF is to convert a video test into a low-dimensional vector by taking into account the temporal continuity of frames.
- **Video Embedding Features (EF)** EF captures the intrinsic information of a given video test input itself. More specifically, EF captures the temporal dimension of video data and is obtained using existing frame sampling techniques (Team 2023) that are specifically designed for video data.
- **Prediction Features (PF)** PF captures the model’s classification information for a test input. PF features are derived from the output of a DNN classifier and represent the confidence of a prediction result, as previously utilized in several studies (Li et al. 2019; Feng et al. 2020).
- **Uncertainty Features (UF)** UF captures the uncertainty associated with the model’s classification. UF features are generated by calculating the uncertainty scores assigned to each test input using existing uncertainty metrics, such as DeepGini (Feng et al. 2020).

VRank demonstrates applicability in various domains. For example, when evaluating a video classification model designed to identify accident videos captured by highway cameras, VRank can be utilized to detect potentially misclassified video test cases within the test dataset. These video tests have a higher likelihood of uncovering bugs in the model. Through early labeling and diagnosis of these video tests, VRank can accelerate the model debugging process, minimizing the need for time and manual labeling efforts.

Moreover, prioritizing video-type test inputs can provide several benefits for developers in the context of DNN testing: **1) Save labeling time and cost:** Prioritizing video data for testing can save the cost of traditional manual labeling. Developers can quickly identify tests that are most likely to be incorrectly predicted by the model and label them, reducing the overall labeling cost. Videos typically contain numerous frames and continuous dynamic information, requiring a significant investment of time and manual effort for labeling. Test prioritization can help reduce the cost of manual labeling; **2) Rapidly uncover bugs in video models:** Test prioritization on video-type tests can help developers quickly identify tests that are more likely to be misclassified by the video classification model. These tests can efficiently aid in identifying bugs in the model; **3) Identify weight parameters causing prediction errors:** These potentially misclassified tests can also assist developers in efficiently analyzing which weight parameters in the model are responsible for causing prediction errors; **4) Fine-tuning of video models:** Through prioritizing video-type tests for rapid bug identification and quick recognition of weight parameters associated with causing prediction errors, developers can better perform model fine-tuning.

We conducted an empirical study to evaluate the performance of VRank based on 120 subjects. Here, a subject refers to a pair of video dataset and DNN model. We compare VRank with four test prioritization approaches compatible with video datasets and one baseline method, random selection. Furthermore, we evaluated the effectiveness of VRank in scenarios where noise is present during testing. Our experimental results demonstrate that VRank achieved better effectiveness over all the compared test prioritization approaches, with an average improvement of 5.76%$\sim$46.51% on natural datasets and 4.26%$\sim$53.56% on noisy datasets. We publish our dataset, results, and tools to the community on Github^1^.

Our work has the following major contributions:

❶: **Approach.** We propose VRank, the first test prioritization approach that is specifically designed for video datasets. Specifically, VRank utilizes video-oriented feature generation and learning-to-rank techniques to rank the test inputs and prioritize potentially-misclassified video inputs.
❷: **Study** We conduct an extensive study involving 120 subjects, including natural and noisy test sets, to evaluate the performance of VRank. We compare VRank against existing test prioritization approaches and random selection. Our experimental results demonstrate the effectiveness of VRank.
❸: **Feature contribution analysis** We conducted a comprehensive analysis to assess the individual contributions of various feature types to the effectiveness of VRank. Our findings demonstrate that all four types of generated features, namely temporal features (TF), uncertainty features (UF), prediction features (PF), and video embedding features (EF), contribute to enhancing the effectiveness of VRank.

The remaining sections of our paper are organized as follows. Section 2 provides the background for our work. Section 3 presents the specific details of the VRank approach we propose. Section 4 exhibits the design of our study. Section 5 presents the relevant details of the experiments and the analysis of the experimental results. Section 6 discusses the limitations and threats to the validity of our study. Section 7 presents the related work of our study. Finally, we conclude our paper in Section 8.

## Background

### DNNs and DNN Testing

Classification deep neural networks (DNNs) (Zeng et al. 2014) are foundational to many applications of deep learning (Li et al. 2022). These networks are characterized by their multilayer architecture consisting of an input layer, one or more hidden layers, and an output layer. Each layer of a DNN comprises a set of interconnected neurons (Liu et al. 2017) that interconnect via weighted edges. A neuron is a computational unit that applies an activation function to the inputs and the weights of the incoming edges. The resulting output is then propagated to the next layer via the edges. During training, the DNN automatically learns the optimal weights of the edges using a large set of labeled training data. Once trained, the DNN can accurately classify an input object, such as an image or a video, into its corresponding class or category.

Ensuring the quality and reliability of DNN models is of paramount importance, and DNN testing (Chen et al. 2020; Feng et al. 2020; Li et al. 2019; Xie et al. 2011; Du et al. 2019; Cheng et al. 2018; Aggarwal et al. 2019) has emerged as a widely used approach to achieve this goal. Analogous to traditional software systems (Do and Rothermel 2006; Henard et al. 2016; Yoo et al. 2009; Di Nardo et al. 2013; Fang et al. 2014), DNN testing involves inputs and oracles. In the context of DNN testing, test inputs refer to the input that the model is expected to classify, which can take diverse forms depending on the specific task of the DNN under test, including images, natural language, or speech. Test oracles in DNN testing rely on manual labeling, whereby each input is manually labeled with ground truth by human annotators. By comparing the labeled ground truth and the predicted output of the DNN model, it is possible to assess the accuracy of the model in predicting the correct output for the given input.

### DNNs for Video Classification

In recent years, the volume of multimedia content available on the Internet has increased exponentially, leading to an explosion in the number of videos being shared every minute. This rapid growth of video content has created a pressing need to analyze and understand these videos for a variety of applications, including search, recommendation, and ranking. Over the past few decades, the computer vision community (Wang et al. 2018) has focused on developing algorithms to address different video analysis problems, notably video classification. While significant progress has been made in feature learning using deep learning approaches in the image domain (Krizhevsky et al. 2017), pre-trained convolutional network (ConvNet) models (Jia et al. 2014) have been developed for generating image features. These features represent the activations of the network’s last few fully-connected layers. However, applying these image-based deep features directly to videos is typically not feasible.

To overcome this issue, Tran et al. (2015) proposed the use of deep 3D ConvNets to learn spatio-temporal features for video classification, leveraging large-scale video datasets. Building upon their previous work, Tran et al. (2017) conducted an empirical ConvNet architecture search to improve spatiotemporal feature learning, which outperformed C3D on several datasets, with faster inference time, smaller model size, and more compact representation. In their subsequent work, Tran et al. (2018) investigated several forms of spatiotemporal convolutions for video analysis and their effects on action recognition. Moreover, Feichtenhofer et al. (2019) proposed the SlowFast network for video recognition, which comprises a Slow pathway for capturing spatial semantics and a Fast pathway for capturing motion at a fine temporal resolution.

**Fig. 1:**
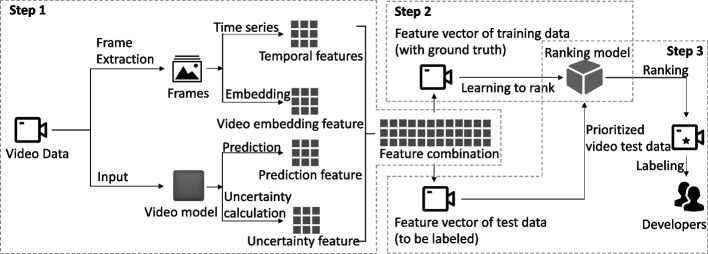
Overview of VRank

### Test Input Prioritization for DNNs

Test input prioritization aims to rank the test inputs based on their likelihood of being incorrectly predicted by a DNN model. The literature has proposed two main categories of approaches for test input prioritization: coverage-based and confidence-based. Coverage-based approaches (e.g., CTM (Yoo and Harman 2012)) extend traditional software system testing methods to DNN testing. The research work conducted by Feng et al. (2020) compared their proposed confidence-based approach DeepGini with numerous coverage-based approaches, demonstrating that DeepGini outperforms existing coverage-based techniques in prioritizing tests regarding both effectiveness and efficiency. Weiss and Tonella (2022) further conducted an extensive investigation of several notable uncertainty-based metrics like Vanilla SM, Prediction-Confidence Score (PCS), and Entropy. These metrics have been demonstrated to be effective in test prioritization. While the aforementioned confidence-based approaches can be adapted to prioritize video test inputs, they fail to account for the distinct characteristics inherent in video data during the test prioritization process. In contrast, our proposed VRank explicitly considers the unique features of videos by utilizing a carefully designed feature generation strategy. By taking into account these video-specific features, VRank achieves higher prioritization effectiveness compared to the aforementioned uncertainty-based methods. Currently, Wang et al. (2021) proposed PRIMA, which is based on mutation analysis and learning-to-rank. However, PRIMA is not applicable to video-oriented test prioritization because PRIMA’s mutation rules are not adapted to video data.

## Approach

### Overview

Figure 1 illustrates the comprehensive outline of the sequential stages involved in our proposed VRank test prioritization approach. In the subsequent sections, we provide a more detailed description of each step.

❶: **Feature vector generation** Given a video test set *T* and the model *M* to be evaluated, in this step, VRank aims to generate a feature vector for each test $t \in T$. To this end, for each test, VRank generates four different types of features for it and combines these features into a final feature vector. The specific methods for feature generation can be found in Section 3.2. Furthermore, Section 3.2 also describes how to combine the generated four different types of features into a final feature vector.
❷: **Ranking model training** After obtaining the final feature vector for each test input $t \in T$, in this step, we aim to leverage a ranking model to predict the probability of each test being predicted incorrectly by the model *M* based on its final feature vector. The specific details regarding the training process of the ranking model and the methodology for utilizing the ranking model to predict misclassification probabilities can be found in Section 3.3.
❸: **Test prioritization** After obtaining the probability of each test (in the test set *T*) being misclassified by the model *M* using the ranking model, VRank utilizes this information for test prioritization. Tests with a higher probability of being misclassified will be prioritized higher. The specific details of this step can be found in Section 3.4.

In the subsequent sections, we provide a comprehensive description of each step outlined aforementioned, encompassing Video-oriented Feature Generation (cf. Section 3.2), Learning-to-rank (cf. Section 3.3), Variants of VRank (cf. Section 3.5), and the Usage of VRank (cf. Section 3.6). These sections delve into the intricate details of each step, offering a thorough understanding of the methodologies employed and their associated considerations.

### Step 1: Video-oriented Feature Generation

Given a test set *T* of videos and a DNN model *M* to be tested, the objective of VRank is to prioritize tests that are more likely to be misclassified by the model *M*. VRank is based on video-oriented feature generation and the learning-to-rank technique. Therefore, in the first step, VRank generates four types of features for each video-type test input. In the following, we provide a comprehensive elucidation of the details for each type of feature, delving into the underlying rationale behind their inclusion in VRank and the methods employed for their generation. We aim to establish a clear understanding of their significance and relevance in the context of VRank.

- **Temporal Features(TF)** TF captures the distinctive temporal coherence within a given video-type test. The primary aim of generating TF is to transform a video test into a low-dimensional vector by considering temporal frame continuity. In the following, we present the two main approaches we employed for generating TF features from consecutive frames: 1) Feature generation based on temporal changes. We compute variations between adjacent frames, encompassing Euclidean distance (Liberti et al. 2014), Manhattan distance (Malkauthekar 2013), squared difference distance (Pillichshammer 2000), and Pearson similarity (Cohen et al. 2009). These metrics can indicate the extent of change between frames, effectively capturing the dynamic information of the video. 2) Statistical feature computation. We calculate statistical features such as variance, mean, and median for consecutive frames. These features contribute to delineating the individual characteristics of each frame.
- **Video Embedding Features (EF)** capture the intrinsic information of a given video test input. To obtain EF, we employ existing frame sampling techniques (Team 2023) to extract a fixed number of frames from a given video-type test input *t*. We then utilize the pre-trained ResNet model (He et al. 2016) to map each frame into a vector representation. Finally, we compute the average of all frame vectors to obtain a representative vector for the entire video.
- **Prediction Features (PF)** captures the model’s classification information for a test input. To obtain PF, we input *t* into the model *M*, and *M* will output a probability vector representing the probabilities of *t* belonging to each class. For example, a feature vector $\{0.2, 0.3, 0.5\}$ signifies that, according to the predictions made by model *M*, the test input *t* has a 20% probability of belonging to class 1, a 30% probability of belonging to class 2, and a 50% probability of belonging to class 3. PF has been utilized in various prior studies (Li et al. 2019; Feng et al. 2020).
- **Uncertainty Features (UF)** captures the uncertainty associated with the model’s classification. To obtain UF, we leverage six existing uncertainty metrics (Weiss and Tonella 2022; Feng et al. 2020; Wang and Shang 2014) (i.e., DeepGini, Vanilla SM, PCS, Entropy, Margin, and Least Confidence) to obtain a set of uncertainty scores for each test input *t*. These metrics have been widely recognized for their outstanding effectiveness in quantifying uncertainty in classification tasks. For each test input *t*, we compute the corresponding uncertainty scores using each of the six metrics. These scores represent the model’s uncertainty in predicting the class for *t*. The UF vector for a given test input is then constructed by concatenating the six uncertainty scores, resulting in a vector representation: $\{$ $S_1$, $S_2$, $S_3$, $S_4$, $S_5$, $S_6$ $\}$. Each element $S_i$ represents the uncertainty associated with the model’s prediction for the test input *t* calculated by the $i_{th}$ uncertainty-based metric.

For each test input $t \in T$, VRank combines its four aforementioned types of features to generate a comprehensive and representative feature vector. This feature vector encapsulates the relevant information from all feature types for the given test input.

Below, we explain how the aforementioned features contribute to determining the decision boundaries of the model:

- **Temporal Features (TF)** Temporal features can capture the unique temporal coherence in a given video type test. Generating time features allows the transformation of video tests into low-dimensional vectors, where the model’s decision boundaries can be perceived as a geometric interface. Low-dimensional video vectors, when mapped into space, can indirectly reflect the distance between the video-type test and the decision boundary.
- **Video Embedding Features (EF)** These features can effectively capture the intrinsic information of the video test input, particularly the temporal dimension of video data. Through this capture, the video input can be mapped to a spatial vector, where the model’s decision boundaries can be seen as a geometric interface. The numerical video embedding feature can facilitate the calculation of the distance between a video-type test and the decision boundary. Therefore, the embedding feature can indirectly reflect the proximity between a test and the decision boundaries.
- **Prediction Features (PF)** These features originate from the DNN classifier’s classification information for the test input. PF features reflect the model’s confidence in the prediction results and can be used to evaluate the model’s accuracy in predicting specific test inputs. If a test input’s PF features indicate that the model is not confident in its classification result, it can suggest that the input is close to the model’s decision boundaries.
- **Uncertainty Features (UF)** These features represent the model’s uncertainty about its classification decisions. By calculating uncertainty scores for each test input (e.g., using DeepGini), UF features can assist in identifying test inputs for which the model exhibits higher uncertainty during classification. Test inputs with high uncertainty are more likely to be located near the model’s decision boundaries.

Below, through a specific example, we illustrate how VRank integrates the aforementioned four types of features into a final feature vector. Assuming that, for a given video-type test input, VRank generates four types of features for it: temporal features (TF) of *i* dimensions, denoted as ($v_1$, $v_2$, ..., $v_i$), embedding features (EF) of *j* dimensions, denoted as ($e_1$, $e_2$, ..., $e_j$), prediction features (PF) of k dimensions, denoted as ($p_1$, $p_2$, ..., $p_k$), and uncertainty features (UF) of *n* dimensions, denoted as ($u_1$, $u_2$, ..., $u_n$). VRank combines these four types of vectors by concatenation, producing a final vector of ($i+j+k+n$) dimensions: ($v_1$, $v_2$, ...$v_i$, $e_1$, $e_2$, ..., $e_j$, $p_1$, $p_2$, ..., $p_k$, $u_1$, $u_2$, ...,$u_n$).

In the following section, we provide a detailed explanation of the methodology employed to obtain the misclassification score.

### Step 2: Learning-to-rank

In this step, we employ the ranking model LightGBM (Ke et al. 2017) to learn from the feature vector of $v \in V$ to predict its misclassification score. LightGBM is an advanced gradient-boosting framework renowned for its ability to learn features for efficient classifications. We follow the process below to train the LightGBM model: Given the video classification *M* with the dataset used for its evaluation, we initially partition the dataset into two sets: the training set *R* and the test set *T*. The test set is kept untouched for evaluating VRank. Our objective is to construct a training set $R'$ for training the ranking models based on the training set *R*. To achieve this, we generate the final feature vector for each $r \in R$ by following the steps in Section 3.2. These features serve as the training features for the dataset *R*. Subsequently, we employ the original video classification model *M* to classify each instance $r \in R$, aiming to identify whether each *r* is misclassified by the model *M*. If *r* is misclassified, it will be labelled as 1; otherwise, it will be labelled as 0. Consequently, we obtain the labels for the training set *R*. Using the constructed training set, we train the LightGBM ranking model for VRank.

### Step 3: Test Prioritization

The LightGBM ranking model, trained in the previous step, was originally designed for binary classification, classifying a given input into one of two classes. However, our objective is to obtain a misclassification probability score for each test input, indicating the likelihood of it being misclassified by the model. To achieve this, we applied specific adjustments to the original LightGBM model: We extract the intermediate value from the model’s output for a given input, which can indicate the misclassification probability. Typically, in the model prediction process, if this intermediate value exceeds a predefined threshold, the input is labeled as “misclassified"; otherwise, it is labeled as “not misclassified". Instead of proceeding with the final classification, we directly employ this intermediate value as the misclassification probability score. A higher score implies a greater probability of the test instance being misclassified. Finally, we rank all tests in the test set *T* in descending order based on their misclassification probability scores.

### Variants of VRank

We investigate the impact of different ranking models on the effectiveness of VRank and propose three variants of VRank, namely $\text {VRank}^X$, $\text {VRank}^R$, and $\text {VRank}^L$. These variants employ the XGBoost (Chen and Guestrin 2016), Random Forest (Breiman 2001), and Logistic Regression (Minka 2003) respectively, as their underlying ranking models. It is important to note that the execution workflow of these variants closely resembles that of VRank, and the sole distinction lies in the selection of ranking models.

Additionally, we also extended the adjustments made to the ranking model LightGBM of VRank to the ranking models of VRank’s variants. Specifically, for a test input, rather than having the ranking models output a binary classification (i.e., whether the test will be predicted incorrectly by the model), we extract the intermediate output to obtain the probability of this test being misclassified. In this way, we can obtain the misclassification score of each test input, which can be utilized for test prioritization. In the following, we provide a detailed explanation of the specific ranking models utilized by each variant of VRank.

- $\textbf{VRank}^\textbf{X}$ In the context of $\text {VRank}^X$, we leverage the XGBoost ranking algorithm (Chen and Guestrin 2016) to predict the misclassification score associated with a given test input, based on its feature vector. XGBoost is a powerful gradient-boosting technique that effectively integrates decision trees to augment prediction accuracy.
- $\textbf{VRank}^\textbf{R}$ In the context $\text {VRank}^R$, we adopt Random Forest (Breiman 2001) as the ranking model. Random forest is an ensemble learning algorithm that constructs multiple decision trees. The predictions from individual trees are combined to produce the final prediction using averaging or voting.
- $\textbf{VRank}^\textbf{L}$ In the context $\text {VRank}^L$, we adopt Logistic Regression (Minka 2003) as the ranking algorithm. Logistic Regression is a statistical modeling technique that uses a logistic function to model the association between a categorical dependent variable and one or more independent variables.

### Usage of VRank

Utilizing ranking models, VRank is capable of predicting a misclassification score for each test input within a designated test set. Test inputs with higher scores are assigned a higher priority. The ranking models employed in VRank undergo pre-training prior to their execution, following standardized and consistent procedures. In the subsequent parts, we comprehensively present the training process, outlining the specific steps taken to train the ranking models.

Given a video dataset and the model *M* under test, the initial step is to partition the dataset into two subsets: the training set *R* and the test set *T*, with a ratio of 7:3 (Nguyen et al. 2021). The test set *T* remains untouched to evaluate the effectiveness of VRank. Based on the training set *R*, our objective is to construct a new training set $R'$ specifically for training the ranking models. Initially, a feature vector $F_v$ is generated for each input $r \in R$. The generation procedures for the feature vector are described in Section 3.2. These feature vectors are then used to construct a new training set $R'$. To obtain the labels for each sample in $R'$, we input $r_i \in R$ into the model *M*. Leveraging the known ground truth of the training set *R* if $r_i$ is incorrectly predicted by model *M*, the label of the corresponding $r'_i \in R'$ is set to 1; otherwise, it is set to 0.

Based on the constructed training set $R'$, we train the ranking models. Upon the completion of the training process, the ranking models are capable of predicting the likelihood of misclassification for a given test input based on its corresponding feature vector.

## Study Design

### Research Questions

Our experimental evaluation answers the research questions below.

- **RQ1: How does VRank perform in prioritizing video test inputs?** We assess the effectiveness and efficiency of VRank and compare it with multiple existing testing prioritization approaches, including DeepGini, Vanilla Softmax, PCS, Entropy, and random selection.
- **RQ2: How does VRank perform on noisy video data?** To evaluate the effectiveness of VRank in noisy contexts, we employ a range of noise generation techniques derived from prior research works (Shorten and Khoshgoftaar 2019; Perez and Wang 2017; Mikołajczyk and Grochowski 2018; Taylor and Nitschke 2018) to generate video datasets with simulated noise. We compare VRank’s effectiveness on these generated noisy datasets with the aforementioned test prioritization approaches to demonstrate its effectiveness.
- **RQ3: What is the impact of different ranking models on the effectiveness of VRank?** Within the learning-to-rank process of VRank, we employed the LightGBM (Ke et al. 2017) ranking algorithm. In this research question, we introduce three variants of VRank by modifying the ranking models to Random Forest (Breiman 2001), XGBoost (Chen and Guestrin 2016), and Logistic Regression (Minka 2003), respectively. By evaluating the effectiveness of VRank and its variants, we aim to explore which ranking algorithm can better utilize the generated features for test prioritization.
- **RQ4: To what extent do each type of features contribute to the effectiveness of VRank? ** In VRank, we generate four distinct types of features from each test input for test prioritization, namely temporal features (TF), video embedding features (EF), prediction features (PF), and uncertainty features (UF), as elaborated in Section 3. In this research question, we focus on comparing the contributions of the three types of features toward the effectiveness of VRank.
- **RQ5: What is the influence of the number of extracted frames on the effectiveness of VRank?** Two critical steps in VRank are to generate video embedding features and temporal features from a given test to predict the likelihood of the test being misclassified. To obtain these two types of features, we utilize established frame sampling techniques (Team 2023) to extract a fixed number of frames from the video-type test input. In this research question, we explore the impact of the number of extracted frames on the effectiveness of VRank.

### Subjects

The effectiveness of VRank and the compared test prioritization approaches (Feng et al. 2020; Weiss and Tonella 2022) was evaluated using a set of 120 subjects, where each subject corresponds to a video dataset with a model. Essential details regarding these subjects are presented in Table 1. Specifically, the “#Videos” column indicates the number of videos in a dataset, while the “Type” column denotes the dataset’s type. “Original” denotes natural data, while other non-original types are abbreviations representing different types of noise. For instance, “HF” indicates Horizontal Flip noise.

Among the 120 subjects, 15 subjects (3 video datasets $\times$ 5 models) were generated using natural datasets, while the remaining 105 subjects were generated using noisy datasets. To generate the noisy datasets, we applied 7 noise generation techniques to each natural dataset, resulting in 7 noisy datasets. Each noisy dataset was then paired with 5 models. Therefore, the total number of subjects is 105 (3 video datasets $\times$ 5 models $\times$ 7 noise generation techniques). In the subsequent section, we present a comprehensive description of the datasets and models employed in our research.

**Table 1:** Video models and datasets

ID	Dataset	# Videos	Model	Type
1	UCF101	13320	C3D	Original, HF, HS, WS, FSN, SR, ZCA, CSR
2	UCF101	13320	R2Plus1D	Original, HF, HS, WS, FSN, SR, ZCA, CSR
3	UCF101	13320	R3D	Original, HF, HS, WS, FSN, SR, ZCA, CSR
4	UCF101	13320	SlowFastNet	Original, HF, HS, WS, FSN, SR, ZCA, CSR
5	UCF101	13320	VT	Original, HF, HS, WS, FSN, SR, ZCA, CSR
6	HMDB51	6849	C3D	Original, HF, HS, WS, FSN, SR, ZCA, CSR
7	HMDB51	6849	R2Plus1D	Original, HF, HS, WS, FSN, SR, ZCA, CSR
8	HMDB51	6849	R3D	Original, HF, HS, WS, FSN, SR, ZCA, CSR
9	HMDB51	6849	SlowFastNet	Original, HF, HS, WS, FSN, SR, ZCA, CSR
10	HMDB51	6849	VT	Original, HF, HS, WS, FSN, SR, ZCA, CSR
11	HWID12	2782	C3D	Original, HF, HS, WS, FSN, SR, ZCA, CSR
12	HWID12	2782	R2Plus1D	Original, HF, HS, WS, FSN, SR, ZCA, CSR
13	HWID12	2782	R3D	Original, HF, HS, WS, FSN, SR, ZCA, CSR
14	HWID12	2782	SlowFastNet	Original, HF, HS, WS, FSN, SR, ZCA, CSR
15	HWID12	2782	VT	Original, HF, HS, WS, FSN, SR, ZCA, CSR

#### DNN Models

We assess the effectiveness of VRank based on five prevalent video classification models: C3D (Tran et al. 2015), R3D (Tran et al. 2017), R2Plus1D (Tran et al. 2018), SlowFast (Feichtenhofer et al. 2019) and VT (Paul 2023). The reason we selected these models for evaluating VRank is that: 1) These models are widely recognized in the field of video classification and have extensive applications in both academia and industry (Tran et al. 2015, 2017, 2018; Feichtenhofer et al. 2019). 2) Each model has its unique architecture and approach to handling video data. 3) Since these models have undergone extensive testing and application on multiple datasets (Tran et al. 2015, 2017, 2018; Feichtenhofer et al. 2019), they provide VRank with a solid benchmark for effectively comparing VRank’s performance across different models.

Although we only conduct tests on these specific models, it is important to note that VRank can be applied to a wide range of video classification models.

- **C3D** (Tran et al. 2015) The C3D (Convolutional 3D) network is an architecture of 3D Convolutional Networks designed to learn spatio-temporal data, particularly in the form of videos. C3D comprises eight convolutional layers, five max-pooling layers, and two fully connected layers, followed by a softmax output layer. C3D’s unique ability to model both appearance and motion information simultaneously is a key factor in its superior performance compared to 2D ConvNet features on various video analysis tasks. This is because videos are inherently spatio-temporal and therefore require specialized architectures capable of extracting and processing information in all three dimensions.
- **R3D** (Tran et al. 2017) The R3D network is a variant of 3D Convolutional Networks, incorporating design elements from both ResNet (He et al. 2016) and C3D architectures. Specifically, R3D leverages residual connections from ResNet, which facilitate the training of DNNs by allowing gradients to flow directly through the network. Additionally, the R3D architecture uses 3D ConvNets to learn spatiotemporal features, making it particularly suited for video-based tasks.
- **R2Plus1D** (Tran et al. 2018) The R2Plus1D architecture effectively addresses the computational complexity associated with action recognition tasks by decomposing the 3D convolutions into a fusion of spatial and temporal convolutions. This decomposition enables more efficient utilization of computational resources compared to fully 3D convolutions.
- **SlowFast** (Feichtenhofer et al. 2019) SlowFast is a video recognition architecture that introduces two pathways, namely the slow pathway and the fast pathway. The slow pathway effectively functions at a reduced frame rate, thereby facilitating the extraction and analysis of spatial semantics pertaining to the video content. Conversely, the fast pathway operates at a significantly higher frame rate, affording the capacity to capture motion nuances with exceptional temporal resolution.
- **VT** (Paul 2023) The Video Classification with Transformers (VT) model is an open-source project officially released by the Keras deep learning framework. It integrates Convolutional Neural Networks (CNNs) and Transformers to enhance video classification capabilities. Specifically, it employs CNNs to extract frame-level features from the video and then feeds these features into a Transformer to capture temporal relationships between different frames. The model is designed to effectively learn spatial-temporal features from video data, enhancing its ability to classify video content accurately. This approach showcases an advanced application of deep learning in video analysis.

#### Datasets

In our study, we assess the performance of VRank using three widely-adopted video datasets: HWID12 (Kezebou et al. 2022), HMDB51 (Kuehne et al. 2011), and UCF101 (Soomro et al. 2012). We selected these three datasets to evaluate VRank due to their representativeness and extensive usage in the field of video classification (Kezebou et al. 2022; Kuehne et al. 2011; Soomro et al. 2012). Specifically, their representativeness is mainly reflected in the following aspects: **1) Diversity.** These three datasets cover a wide range of activities and scenes. For example, HWID12 includes real-world surveillance videos of high-speed highway traffic, HMDB51 covers various daily actions, and UCF101 contains a variety of action videos from the real world. This diversity allows these datasets to comprehensively test the performance of video-oriented test prioritization approaches in different contexts. **2) Complexity.** The actions and scenes in the videos have different levels of complexity, including both simple daily activities and complex sports and interactions. This allows for a more representative and comprehensive evaluation of video-oriented test prioritization methods. **3) Widespread Use.** These datasets are widely utilized in both academia and industry (Wang and Schmid 2013; Kuehne et al. 2011), serving as standard benchmarks for many research works.

Although real-world autonomous driving datasets also contain valuable video data, we did not use autonomous driving datasets to evaluate VRank for the following reasons: **1) Difference in Tasks and Application Scenarios.** VRank is primarily designed for DNN models used in video classification tasks. The core of video classification is to categorize a video into a specific label. In other words, video classification models aim to classify a video into a single category based on its content, such as classifying a video as “High Jump", “Diving", etc. In contrast, the main goal of autonomous driving datasets is to enhance the reliability and safety of autonomous driving systems through the detection and tracking of multiple objects. In the context of autonomous driving, a video typically contains multiple labels. For instance, a video clip can simultaneously include dynamic elements such as vehicles, pedestrians, traffic signs, etc. **2) Differences in Dataset Annotation.** The annotation method for autonomous driving datasets is typically frame-based, where various objects in each frame of the image are required to be labeled. Therefore, each video has multiple labels corresponding to the various detected objects. In contrast, VRank is designed for video classification datasets, where the annotation is generally based on the overall video rather than frame-by-frame annotation. In this context, each video has a unique label as a whole.

- **HWID12** (Kezebou et al. 2022) The HWID12 dataset serves for the classification task of real-time highway accident detection in intelligent transportation systems. HWID12 comprises 2,782 video clips with duration ranging from 3 to 8 seconds, categorized into twelve classes (e.g., “Sideswipe collision”, “Collision with motorcycle” and “Pedestrian hit”).
- **HMDB51** (Kuehne et al. 2011) The HMDB51 dataset comprises video clips sourced from movies, supplemented by a small portion obtained from public databases such as the Prelinger Archives, YouTube, and Google Videos. HMDB51 is composed of 6,849 videos, classified into 51 action categories (e.g., “Drink”, “Hug”, and “Walk”), with each category containing at least 100 clips.
- **UCF101** (Soomro et al. 2012) The UCF101 dataset is an action recognition dataset collected from YouTube. UCF101 consists of 13,320 videos, categorized into 101 action classes (e.g., “High Jump”, “Punch”, and “Diving”).

### Noise Generation Techniques

In our study, we employed seven noise generation techniques to generate video inputs with noise. These techniques were selected based on prior research studies (Shorten and Khoshgoftaar 2019; Perez and Wang 2017; Mikołajczyk and Grochowski 2018; Taylor and Nitschke 2018). The following is a description of each technique:

- **Channel Shift (CSR):** CSR applies modifications to the overall color representation of a video by shifting the value of the color channel. This technique introduces color perturbations by adding random noise to each pixel’s color channel values, thus altering the color appearance of the video.
- **Feature-wise Normalization (FSN):** FSN performs normalization of the features in each video input by dividing it with the standard deviation. This process aims to decentralize the video dataset and normalize the feature distributions, enabling the model to capture finer-grained variations in the data.
- **Height Shift (HS):** HS vertically displaces a given video by a certain number of pixels, effectively shifting its position up or down within the frame. This augmentation technique introduces spatial transformations, such as simulating camera movements or object repositioning, by adding random noise to the vertical position of each frame.
- **Width Shift (WS):** WS horizontally shifts the position of a video input by a specified number of pixels. By applying random horizontal offsets to each frame, WS enables the model to learn robustness to variations in object positioning and enhances its ability to handle objects appearing at different spatial locations within the frame.
- **Shear (SR):** SR refers to the intentional distortion of a video along its axes with the primary objective of creating or correcting perceptual angles.
- **Horizontal Flip (HF):** HF horizontally flips a given video by mirroring the content along the vertical axis. This operation introduces left-right orientation changes to the video frames, augmenting the dataset with horizontally flipped versions of the original videos.
- **ZCA Whitening (ZCA):** ZCA whitening applies dimension reduction operations to the given videos, reducing redundant information while preserving crucial features. By performing a linear transformation on the pixel values of each frame, ZCA whitening removes correlations between neighboring pixels, effectively decorrelating the data and enhancing the model’s ability to focus on meaningful variations in the video content.

### Compared Approaches

To demonstrate the effectiveness of VRank, we compare it with five distinct test prioritization approaches, including a baseline approach, namely random selection, alongside four DNN test prioritization techniques. The rationale behind selecting these particular methods rests on three key factors: Firstly, their adaptability to facilitate test prioritization on video datasets, which is a pivotal requirement for our research context. Secondly, their effectiveness in the context of DNNs has been well demonstrated in the existing literature (Feng et al. 2020; Weiss and Tonella 2022; Hu et al. 2021). Lastly, the availability of open-source implementations. All of the selected approaches are accessible for implementation purposes.

- **DeepGini** (Feng et al. 2020) employs the Gini coefficient to measure the likelihood of misclassification, thereby enabling the ranking of test inputs. The calculation of Gini score is presented in Formula (1).
  1 $$\begin{aligned} \xi (x) = 1-\sum _{i=1}^N\left( p_i(x)\right) ^2 \end{aligned}$$
  where $\xi (x)$ refers to the likelihood of the test input *x* being misclassified. $p_i(x)$ refers to the probability that the test input *x* is predicted to be label *i*. *N* refers to the number of labels.
- **Vanilla SM** (Weiss and Tonella 2022) calculates the difference between the value of 1 and the maximum activation probability in the output softmax layer. Formula (2) provides a clear depiction of the calculation process.
  2 $$\begin{aligned} \text{ V }(x)=1-\max _{c=1}^C l_c(x) \end{aligned}$$
  where $l_c(x)$ belongs to a valid softmax array in which all values are between 0 and 1, and their sum is 1.
- **Prediction-Confidence Score (PCS)** PCS (Weiss and Tonella 2022) quantifies the level of uncertainty in a classification model’s prediction for a given test by computing the difference between the predicted class and the second most confident class. PCS is calculated by Formula (3).
  3 $$\begin{aligned} P(x)=l_{k}(x)-l_{j}(x) \end{aligned}$$
  where $l_{k}(x)$ refers to the most confident prediction probability. $l_{j}(x)$ refers to the second most confident prediction probability.
- **Entropy** Entropy (Weiss and Tonella 2022) measures uncertainty in a classification model’s prediction for a given test by computing the entropy of the softmax likelihood.
- **Random selection** (Elbaum et al. 2002) In random selection, the order of execution for test inputs is determined randomly.

### Measurements

Following the prior research on DNN test prioritization (Feng et al. 2020), we employ the  Average Percentage of Fault-Detection (APFD) (Yoo and Harman 2012) metric to assess the effectiveness of VRank and the compared approaches. APFD is a well-established and widely accepted measure for evaluating prioritization strategies. Generally, higher APFD scores indicate a faster rate of misclassification detection. We determine the APFD values by utilizing Formula (4).

4 $$\begin{aligned} A P F D =1-\frac{\sum _{i=1}^k o_i}{k n}+\frac{1}{2 n} \end{aligned}$$

where *n* is the number of test inputs in the test set *T*. *k* is the number of test inputs in *T* that will be misclassified by the DNN model *M*. $o_i$ is the index of the $i_{th}$ misclassified tests in the prioritized test set. More specifically, $o_i$ is an integer that represents the position of the $i_{th}$ misclassified tests in the test set that has been prioritized. Below, we explain the rationale for using APFD to assess the effectiveness of a test prioritization method in detecting misclassified tests: In the formula of APFD (Formula (4)), a smaller $\sum _{i=1}^k o_i$ suggests that the misclassified tests are positioned relatively closer to the front of the prioritized test set. This implies that the prioritization approach effectively places misclassified tests at the beginning of the test set, indicating a higher level of effectiveness. Consistent with previous research (Feng et al. 2020), we normalize the APFD values to the range [0,1]. A prioritization approach is deemed more effective when the APFD value is closer to 1.

Efficiency measurement of VRank: Following the existing study (Wang et al. 2021), we evaluate the efficiency of VRank by quantifying the time required for each step of VRank, as well as the time cost of each compared approach.

### Implementation and Configuration

VRank was implemented in Python utilizing PyTorch 2.0.0 (Paszke et al. 2019), OpenCV 4.7.0, and scikit-learn 1.0.2 libraries. In terms of the compared approaches (Feng et al. 2020; Weiss and Tonella 2022), we integrated existing implementations of them into our experimental pipeline. In terms of ranking models, for XGBoost and LightGBM, we employed the specific versions XGBoost 1.7.4 and LightGBM 3.3.5. For the ranking model random forest and logistic regression, we leveraged the existing algorithm packages provided by scikit-learn. Concerning the parameter configurations, we set the $n\_estimators$ parameter to 100 for the XGBoost, LightGBM, and Random Forest ranking algorithms. For the Logistic Regression ranking algorithm, we set the $max\_iter$ parameter to 100. Our experiments were conducted on NVIDIA Tesla V100 32GB GPUs. In terms of data analysis, the corresponding experiments were performed on a MacBook Pro laptop with Mac OS Big Sur 11.6, Intel Core i9 CPU, and 64 GB RAM.

## Results and Analysis

### RQ1: Effectiveness and Efficiency of VRank

**Objective:** We investigate the effectiveness and efficiency of VRank, comparing it with existing test input prioritization approaches and random selection.

**Experimental design:** We employed 12 pairs of video datasets and models as subjects in our study to assess the effectiveness of VRank. The fundamental details of these datasets and models can be found in Table 1. Specifically, we selected five compared approaches, consisting of four test prioritization techniques (namely DeepGini, Vanilla SM, PCS, and Entropy) along with a baseline approach (random selection). We utilize these approaches for comparison because they can be adapted to prioritize testing on video datasets. To quantify the effectiveness of each approach, we employed the Average Percentage of Fault-Detection (APFD), a widely accepted measure in the field. In addition to assessing effectiveness, we investigated the efficiency of VRank by analyzing the time required for each step of its execution and comparing its overall execution time with that of the five compared approaches.

Furthermore, due to the randomness of the model training process, we performed a statistical analysis to ensure the stability of our findings. Specifically, we repeated all experiments ten times for each subject and reported the average results. Furthermore, we calculated the p-value of the experiments to assess whether the VRank approach consistently outperformed the compared approaches.

To further illustrate the statistical significance of the improvements in VRank compared to other test prioritization approaches, we conducted a statistical analysis by calculating p-values and effect size associated with the experimental results. Regarding the calculation of p-values, we employed the **paired two-sample t-test** (Kim 2015), which is a widely used statistical method for evaluating differences between two related datasets. If the p-value is less than $10^{-05}$, it indicates that the difference between the two sets of data is statistically significant (Ma et al. 2021). For the measurement of effect size, we utilized Cohen’s *d* for measuring the effect size (Kelley and Preacher 2012). In this context, values of $|d|<0.2$ are categorized as “negligible,” $|d|<0.5$ as “small,” $|d|<0.8$ as “medium,” and otherwise as “large”. For instance, if we compare VRank with another test prioritization method, and the value of *d* is 0.7, the effect size is categorized as “medium” because 0.5 < 0.7 < 0.8. This suggests that there is a relatively medium difference between the two methods.

**Table 2:** Effectiveness comparison among VRank, Random, DeepGini, VanillaSM, PCS, and Entropy in terms of the APFD values on natural datasets

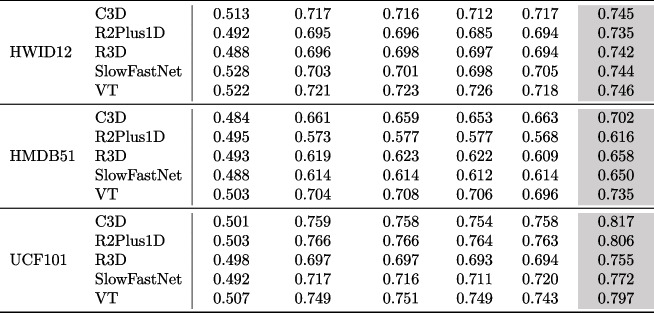

**Table 3:** Performance improvement of VRank on the 15 initial subjects (i.e., three natural input sets on 5 Video classification models)

Approach	# Best cases	Average APFD	Improvement(%)
Random	0	0.501	46.51
DeepGini	0	0.692	6.07
VanillaSM	0	0.694	5.76
PCS	0	0.691	6.22
Entropy	0	0.690	6.38
VRank	15	0.734	−

**Results:** The experimental findings pertaining to RQ1 are presented in Tables 2, 3, 4 and 5. We highlight the approach with the highest effectiveness in grey to facilitate quick and easy interpretation of the results. Table 2 presents the effectiveness of VRank and the compared approaches across different video subjects, as measured by the Average Percentage of Faults Detected (APFD). We see that VRank performs better than all the compared approaches regarding APFD across all cases. Specifically, the APFD range for VRank spans from 0.616 to 0.817, whereas the baseline approach exhibits an APFD range of 0.484 to 0.528. Furthermore, the uncertainty-based test prioritization methods yield an APFD range of 0.563 to 0.766. Table 3 provides a detailed analysis of the experimental results for RQ1, focusing on three aspects: the number of best-performing cases for each test prioritization approach, the average effectiveness, and the relative improvement of VRank compared to each method. It is observed that the average APFD of VRank is 0.734, with an average improvement of 5.76%$\sim$46.51% compared to the uncertainty-based test prioritization approaches and random selection. Based on the aforementioned results, we conclude that VRank exhibits better effectiveness in prioritizing video test inputs compared to DeepGini, VanillaSM, PCS, Entropy, and Random Selection.

**Table 4:** Statistical analysis on natural test inputs (in terms of p-value and effect size)

	Random	DeepGini	VanillaSM	PCS	Entropy
VRank (p-value)	$1.755 \times 10^{-09}$	$7.336 \times 10^{-07}$	$1.023 \times 10^{-06}$	$1.605 \times 10^{-06}$	$6.311 \times 10^{-07}$
VRank (effect size)	7.615	3.816	3.669	3.479	3.884

**Table 5:** Time cost of VRank and the compared approaches

Time cost	Approach					
	VRank	Random	DeepGini	VanillaSM	PCS	Entropy
Feature generation	2.3 min	–	–	–	–	–
Ranking model training	35 s	–	–	–	–	–
Prediction	$<1$ s	$<1$ s	$<1$ s	$<1$ s	$<1$ s	$<1$ s

**Table 6:** Overall effectiveness comparison on noisy video datasets

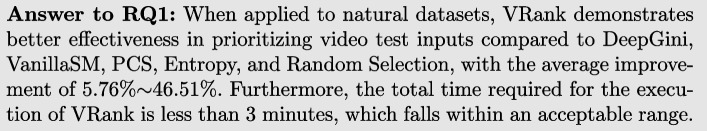

**Table 7:** Performance improvement of VRank on the 105 noisy subjects (i.e., 3(natural input sets)$\times$5(Video classification models)$\times$7(noise technique)

Approach	# Best cases	Average APFD	Improvement(%)
Random	0	0.499	38.68
DeepGini	0	0.645	7.29
VanillaSM	0	0.646	7.12
PCS	0	0.642	7.79
Entropy	0	0.644	7.45
VRank	105	0.692	–

Table 4 presents the results of the statistical analysis evaluating the improvement of VRank in comparison to other test prioritization methods. The analysis employs two key metrics: p-value and effect size. As mentioned in the experimental design above, a p-value below $10^{-05}$ indicates that the difference between two datasets (Ma et al. 2021) is statistically significant. An effect size of $\ge$ 0.8 suggests that the difference in effectiveness between the two approaches is considered “large”.

In Table 4, we see that all the p-values between VRank and other test prioritization approaches consistently fall below $10^{-05}$. This suggests that VRank significantly outperforms all the test prioritization methods being compared. For example, the p-value between VRank and DeepGini is $7.336 \times 10^{-07}$, while the p-value between VRank and VanillaSM is $1.023 \times 10^{-06}$. Moreover, the effect sizes between VRank and all the compared approaches exceed 0.8, suggesting that the improvement in VRank’s effectiveness (measured by APFD) compared to all the other approaches is “large”. For instance, the effect size between VRank and VanillaSM is 3.669, and the effect size between VRank and DeepGini is 3.816.

Table 5 provides a comprehensive breakdown of the time required by each step of VRank and a comparison with uncertainty-based test prioritization approaches and random selection. The time required for VRank is partitioned into three steps: feature generation, ranking model training, and prediction. Our findings reveal that feature generation is the most time-consuming step, taking approximately 2.3 minutes, followed by ranking model training, which takes approximately 35 seconds. Notably, the prediction time of VRank is fast, taking less than 1 second once the ranking model is trained and the features have been generated. Overall, the average time consumption of VRank for each dataset is approximately 3 minutes. Although VRank is less efficient than uncertainty-based test prioritization approaches, which take less than 1 second, its time cost is acceptable compared to the prohibitively expensive manual labeling.

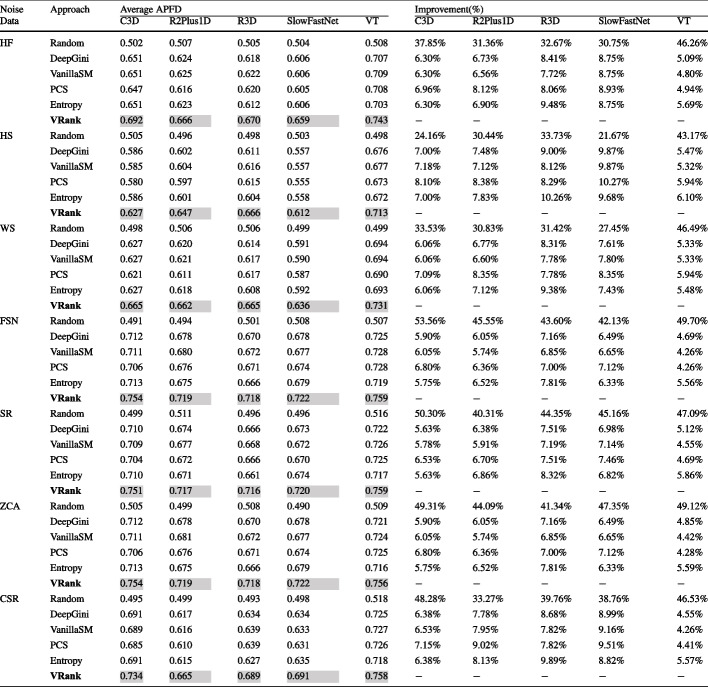



### RQ2: Effectiveness on Noisy Test Inputs

**Objective** We evaluated the effectiveness of VRank on noisy test inputs. To this end, we incorporated various types of video noise, namely Channel Shift (CSR), Feature-wise Normalization (FSN), Height Shift (HS), Width Shift (WS), Shear (SR), Horizontal Flip (HF), and ZCA Whitening (ZCA), as discussed in Section 4.3. We derived inspiration for these noise types from prior research (Shorten and Khoshgoftaar 2019; Perez and Wang 2017; Mikołajczyk and Grochowski 2018; Taylor and Nitschke 2018).

**Experimental Design** In order to generate noisy video datasets, we employed seven noise generation techniques, namely Channel Shift (CSR), Feature-wise Normalization (FSN), Height Shift (HS), Width Shift (WS), Shear (SR), Horizontal Flip (HF), and ZCA Whitening (ZCA). By applying these techniques, we introduced various forms of noise and perturbations to the original video datasets, thereby increasing their diversity and complexity. In total, we constructed 84 subjects for evaluation (4 video models $\times$ 3 video datasets $\times$ 7 noise generation techniques). Consistent with our previous research question, we compared VRank with four test prioritization approaches and a baseline method (i.e., random selection), using the metric APFD to quantify their effectiveness.

**Results** The experimental results for RQ2 are presented in Tables 6, 7 and 8. Table 6 showcases the effectiveness of VRank in comparison to several test prioritization techniques and the baseline (i.e., random selection) across noisy datasets generated using various noise generation techniques. We see that VRank consistently performs better than all the compared methods in terms of average APFD across all cases. More specifically, the average APFD of VRank ranges from 0.612 to 0.758, while the baseline method exhibits an average APFD ranging from 0.490 to 0.518. The uncertainty-based test prioritization techniques achieve an average APFD between 0.555 to 0.728. Overall, VRank demonstrates an improvement ranging from 4.26% to 53.56% compared with DeepGini, VanillaSM, PCS, Entropy, and Random Selection. This improvement is consistently observed across each noise generation technique. For instance, under the HS noise technique, VRank exhibits an improvement ranging from 5.32% to 43.17%. Similarly, under the HF noise technique, the improvement ranges from 4.80% to 46.26%, and under the CSR noise technique, it ranges from 4.26% to 48.28%.

Table 7 provides a detailed analysis of the experimental results for RQ2, focusing on three aspects: the number of best-performing cases for each test prioritization approach, the average effectiveness, and the relative improvement of VRank compared to each method. We see the average APFD of VRank is 0.692, with an average improvement of 7.12% to 38.68% compared to other test prioritization approaches.

In Table 8, we present a detailed analysis of VRank’s effectiveness by using the SR noise technique as an example. We can see that VRank consistently performs better than the compared approaches across all subjects (a DNN model associated with a noisy dataset) related to SR. Moreover, the APFD values of VRank range from 0.577 to 0.756, while that of the compared approaches range from 0.496 to 0.712. The aforementioned experimental results indicate that VRank maintains better effectiveness over all the compared approaches on noisy video datasets. 
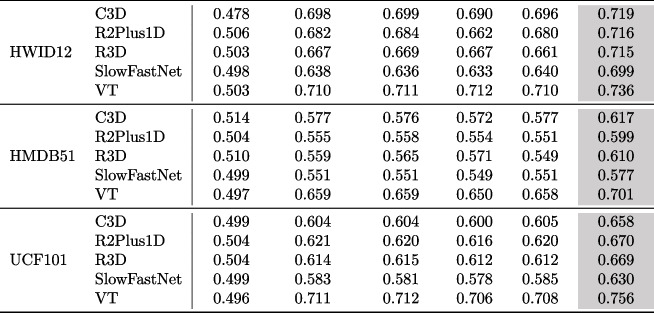


**Table 8:** Effectiveness comparison on noisy datasets generated by the WS noise generation technique

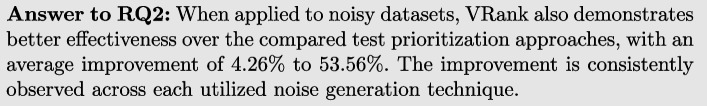	

### RQ3: Impact of Different Ranking Models

**Objective** We explore the efficacy of various ranking models in VRank concerning their ability to leverage the generated video features for test prioritization.

**Experimental Design** In order to explore the influence of different ranking models on the effectiveness of VRank, we have proposed three VRank variants that employ different ranking models for the learning-to-rank process. Specifically, we evaluate VRank along with its variants, namely $\text {VRank}^X$, $\text {VRank}^R$, and $\text {VRank}^L$ (as described in Section 3.5), on their ability to prioritize test inputs in both natural and noisy settings and assess their effectiveness in terms of APFD.

**Table 9:** Performance (APFD scores) of VRank variants with different ranking models (#BC $\Leftrightarrow$ #Best cases) and (Avg $\Leftrightarrow$ Average APFD score)

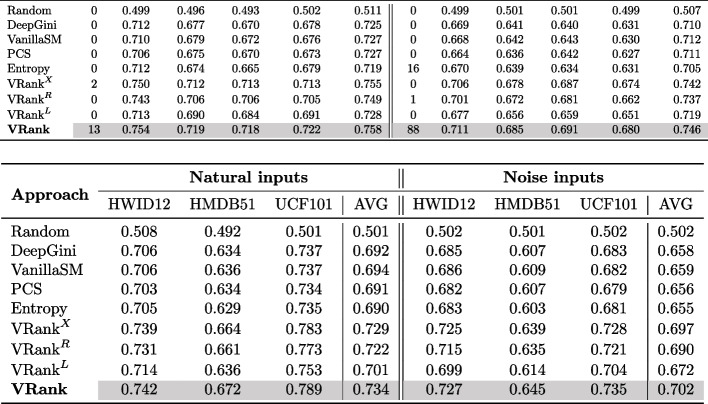	

**Results** The experimental results pertaining to RQ3 are presented in Table 9. The upper segment of the table displays the average effectiveness across diverse models, while the lower segment showcases the average effectiveness across different video datasets. We see that both VRank and its variants perform better than all the compared approaches on average. Specifically, in the case of natural inputs, VRank exhibits the highest performance in 86.67% of instances, whereas $\text {VRank}^X$ achieves superiority in the remaining 13.33% of cases. In the context of noisy data, VRank achieves the highest performance in 84.61% of cases, while $\text {VRank}^X$ excels in the remaining 15.38% of cases. Furthermore, the mean APFD values for VRank and its variants on the natural dataset range from 0.718 to 0.758, while the compared approaches exhibit mean APFD values ranging from 0.493 to 0.755. On the noisy dataset, the mean APFD values for VRank and its variants range from 0.680 to 0.746, while the compared methods exhibit mean APFD values ranging from 0.499 to 0.742. These findings demonstrate that VRank and its variants perform better than all the compared methods on average.

Furthermore, we see that VRank demonstrates the highest effectiveness among all variants. As shown in Table 9, regardless of whether the datasets are natural or noisy, VRank consistently achieves the highest average APFD across all cases. In the natural dataset scenario, the mean APFD of VRank reaches 0.734, while the variants exhibit mean values ranging from 0.701 to 0.729. In the noisy dataset scenario, VRank attains an average APFD of 0.702, whereas the variants exhibit mean values ranging from 0.672 to 0.697. These results indicate that VRank surpasses all variants in terms of effectiveness, suggesting that the ranking model employed by VRank, namely LightGBM, outperforms the ranking models utilized by the variants in leveraging the features of video input for test prioritization.

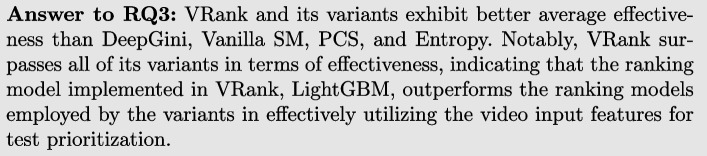



### RQ4: Feature Contribution Analysis

**Objective** We aim to investigate the contributions of different types of features to the effectiveness of VRank.

**Experimental Design** To evaluate the importance of different types of features for VRank, we leverage the cover metric in the XGBoost algorithm (Chen and Guestrin 2016). The cover metric provides a means of evaluating feature importance by quantifying the average coverage of each instance through the leaf nodes within a decision tree. Specifically, this metric entails the calculation of the frequency with which a specific feature is employed for partitioning the data across all trees within the ensemble, followed by the summation of the coverage values associated with each feature across all trees. Subsequently, the resulting coverage value is appropriately normalized by the total number of instances, thereby yielding the average coverage of each instance by the leaf nodes. The significance of a particular feature is then ascertained based on its derived coverage value, with features exhibiting higher coverage values being attributed greater importance. Upon computing the importance scores for all features, the identification of the top-N important features was carried out for each dataset, thereby providing an elucidation of the feature types that significantly contribute to the effectiveness of VRank.

Moreover, in order to assess the impact of each feature type on the effectiveness of VRank, we conducted a carefully designed ablation study following the methodology outlined in prior research (Du 2020). More specifically, we removed individual feature types and evaluated VRank’s effectiveness under these modified conditions. For example, to measure the contribution of UF features, VRank was executed with UF features excluded while retaining the other three feature types. The resulting performance of VRank was then evaluated under these adjusted circumstances. Similarly, to evaluate the contribution of EF features, VRank was executed without generating EF features while still generating the other three feature types. The performance of VRank was subsequently assessed in this context. Through the conducted ablation study, we can compare the contribution of each feature type to the overall effectiveness of VRank.

**Table 10:** Top-10 features in terms of the average contribution

Rank	HWID12		HMDB51		UCF101	
	Feature	Score	Feature	Score	Feature	Score
1	$\textrm{UF}^5$	88.56	$\textrm{UF}^0$	104.31	$\textrm{UF}^1$	280.18
2	$\textrm{UF}^1$	80.86	$\textrm{EF}^{1352}$	84.75	$\textrm{EF}^{984}$	248.26
3	$\textrm{PF}^{11}$	61.34	$\textrm{TF}^{2070}$	80.48	$\textrm{EF}^{1386}$	219.73
4	$\textrm{EF}^{52}$	38.23	$\textrm{EF}^{443}$	72.76	$\textrm{PF}^{65}$	211.38
5	$\textrm{EF}^{2048}$	37.49	$\textrm{UF}^{2}$	70.74	$\textrm{TF}^{2153}$	201.27
6	$\textrm{TF}^{2104}$	37.48	$\textrm{UF}^{1}$	68.38	$\textrm{UF}^5$	163.01
7	$\textrm{EF}^{1456}$	36.43	$\textrm{EF}^{1819}$	67.33	$\textrm{TF}^{2164}$	154.78
8	$\textrm{TF}^{2113}$	34.88	$\textrm{TF}^{2115}$	64.25	$\textrm{PF}^{18}$	147.84
9	$\textrm{TF}^{2068}$	33.43	$\textrm{EF}^{2124}$	64.06	$\textrm{TF}^{2184}$	146.92
10	$\textrm{PF}^{7}$	32.13	$\textrm{PF}^{44}$	63.45	$\textrm{UF}^5$	139.84

**Table 11:** Ablation study on different features of VRank: Embedding Features(EF), Temporal Features(TF), Prediction Features(PF), Uncertainty Features(UF). ‘w/o’ means ‘without’

Approach	Dataset			Average
	HWID12	HMDB51	UCF101	
VRank w/o EF	0.727	0.656	0.772	0.718
VRank w/o TF	0.731	0.654	0.771	0.719
VRank w/o PF	0.729	0.653	0.768	0.717
VRank w/o UF	0.732	0.652	0.763	0.715
VRank				

**Results** The findings for RQ4 are presented in Table 10. In Table 10, the abbreviations UF, PF, EF, and TF represent uncertainty-based features, prediction features, video embedding features, and temporal features. Additionally, the small superscript numbers on the upper right corner of the feature abbreviations indicate the index of the feature. For instance, $\textrm{UF}^5$ denotes the UF feature with index 5. In Table 10, we see that, across different video datasets (i.e., HWID12, HMDB51, and UCF101), all four types of features appear among the top 10 most contributing features. Specifically, for the HWID12 dataset, UF features contribute to 20% of the top 10 features, PF features contribute to 20%, EF features contribute to 30%, and TF features contribute to 30%. In the case of the HMDB51 dataset, UF, PF, EF, and TF features contribute 30%, 10%, 40%, and 20%, respectively. These experimental results illustrate that all four types of generated features make visible contributions to the effectiveness of VRank.

The experimental results of the ablation study are presented in Table 11. In this table, ‘w/o’ stands for ‘without.’ For example, ‘VRank w/o EF’ refers to executing VRank without generating the video embedding features. From Table 11, we see that the original VRank achieves the highest average effectiveness. Removing any type of feature results in a decrease in the effectiveness of VRank, demonstrating that each type of feature contributes to VRank’s effectiveness. For instance, on the HWID12 dataset, the average APFD value of the original VRank is 0.742. Removing video embedding features results in a decline of VRank’s average APFD to 0.727, while the removal of temporal features causes a decrease to 0.731, prediction features to 0.729, and uncertainty features to 0.732.

From Table 11, we see that across different datasets, all four types of features contribute to VRank. Specifically, the average APFD decrease resulting from the removal of EF is 0.016. Removing TF leads to an average APFD decrease of 0.015, while PF removal results in an average APFD decrease of 0.017, and UF removal causes an average APFD decrease of 0.019. These differences are small. Moreover, taking the HMDB51 dataset as an example, the APFD decreases caused by removing the four types of features are 0.016, 0.018, 0.019, and 0.02, respectively. These experimental results suggest that all types of generated features contribute to the effectiveness of VRank.

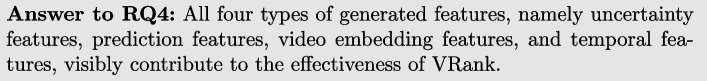



**Table 12:** Influence of the number of extracted frames on the effectiveness of VRank

Data	Frames-4	Frames-8	Frames-16	Frames-32
HWID12	0.724	0.735	0.742	0.748
HMDB51	0.651	0.663	0.672	0.681
UCF101	0.752	0.768	0.789	0.795

### Impact of the Number of Extracted Frames on the Effectiveness of VRank

**Objective** In VRank, two critical steps involve generating video embedding features (EF) and temporal features (TF) from the video-type test to predict the likelihood of the test being misclassified. To obtain EF and TF, we utilize established frame sampling techniques (Team 2023) to extract a fixed number of frames from the video-type test input. In this research question, we explore the impact of the number of extracted frames on the effectiveness of VRank.

**Experimental design** In the original VRank implementation, we extracted 16 frames during the generation of video embedding features and temporal features. To investigate the impact of the number of generated frames, we kept the other execution processes of VRank unchanged and only varied the number of frames extracted, specifically changing it to 4, 8, and 32 frames. The reason we chose these specific numbers of frames to extract is as follows: Selecting 4, 8, and 32 frames can cover a range of frame numbers from relatively low (4 frames) to relatively high (32 frames). This can assist researchers in understanding the impact of different frame count levels on the performance of VRank. We compared the effectiveness (measured by APFD) of VRank with the different number of frames generated. Through comparison, we aim to explore the impact of the number of extracted frames on the effectiveness of VRank.

**Results** The results for RQ5 are presented in Table 12. Specifically, Frames-4 indicates that, during the video embedding feature and temporal feature generation step in VRank, four frames were extracted. Similarly, Frames-8, Frames-16, and Frames-32 correspond to the extraction of 8, 16, and 32 frames, respectively. From Table 12, we see that the effectiveness of VRank increases slightly with the number of extracted frames. In the HWID12 dataset, the effectiveness of VRank with 4 frames to 32 frames is as follows: 0.725, 0.735, 0.742, and 0.748. In the HMDB51 dataset, the effectiveness of VRank with 4 frames to 32 frames is 0.651, 0.663, 0.672, and 0.681. For the UCF101 dataset, the values are 0.752, 0.768, 0.789, and 0.795. We see that on each dataset, VRank’s APFD values gradually improve with an increase in the number of frames. However, the augmentation of frames affects the running time of VRank, impacting efficiency. The original VRank, which utilizes 16 extracted frames, has outperformed all the compared test prioritization methods, and the total execution time is only around 3 minutes. As shown in Table 3, the original VRank (16 frames) outperforms all the compared methods in all cases, with improvements ranging from 5.76% to 46.51%. Therefore, for a trade-off between efficiency and effectiveness, we select to extract 16 frames in the process of generating video embedding features and temporal features. 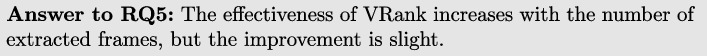


## Discussion

### Limitations

[*Dependency on Visual Features*] One noteworthy limitation of the current implementation of VRank is its exclusive emphasis on extracting visual information from video data, neglecting the incorporation of speech or audio information. This singular focus on visual features hampers the comprehensive understanding of video content, as audio analysis plays a pivotal role in decoding the complete semantic meaning embedded within videos. The absence of audio analysis restricts the model’s ability to capture important auditory cues, such as spoken dialogue, sound effects, or background music, which are integral components of video content. Consequently, the lack of audio analysis may impede the accuracy and effectiveness of the ranking process, as the model’s comprehension of videos remains incomplete and insufficiently nuanced. To address this limitation, future iterations of VRank will include a robust audio analysis component, which will facilitate a more holistic and comprehensive approach to video ranking by encompassing both visual and auditory information. By incorporating audio analysis, VRank will be empowered to leverage the complementary nature of audio-visual data, enabling a more nuanced understanding of video content and enhancing the accuracy and reliability of the ranking process.

[*Contextual Understanding*] While VRank excels in the analysis of individual frames within a video, it can exhibit limitations in comprehending the broader contextual aspects and narrative structure inherent in video content. As focusing solely on individual frames, VRank can lack the temporal relationships and dependencies between frames, thus failing to capture the temporal dynamics and sequential nature of video content. This limitation can pose challenges in accurately ranking videos that heavily rely on temporal coherence, as well as those that require a comprehensive understanding of the entire video content as a cohesive narrative. The lack of contextual understanding may result in an incomplete representation of the video’s meaning and can impact the effectiveness of the ranking process, particularly for videos with intricate storytelling or complex visual narratives. To mitigate this limitation, future research efforts will seek to enhance VRank’s contextual understanding capabilities by exploring methods that can capture narrative structures within videos. By incorporating contextual understanding, VRank will be better equipped to rank videos that exhibit nuanced temporal dynamics, thereby improving its overall performance and applicability in real-world scenarios.

[*Whole Video Classification*] Our research is centered around multi-class datasets that concentrate on classifying entire videos rather than categorizing each frame of a video individually. Specifically, in the video dataset we evaluated, each video (sample) is assigned to a specific category. This implies that within the evaluated video dataset, each frame belongs to the same category. For instance, in the UCF101 dataset, there are a total of 101 categories. A video sample classified as “High Jump” has each frame assigned to the “High Jump” category.

### Threats to Validity

*Threats to Internal Validity.* The internal threats to validity primarily reside within the implementation of our proposed VRank framework and the test prioritization approaches utilized for comparison. To mitigate these threats, we took several measures to ensure the reliability and consistency of our experimental setup. Firstly, we implemented VRank using the widely recognized and extensively utilized PyTorch library, known for its robustness and computational efficiency in deep learning research. By leveraging a well-established framework, we aimed to minimize potential implementation biases. Furthermore, to guarantee the reliability of our comparative analysis, we employed the publicly available implementations of the compared approaches as provided by their respective authors. This approach ensures consistency across the experimental procedures, reducing the risk of implementation discrepancies and increasing the reproducibility of our findings. Another potential internal threat arises from the inherent randomness associated with the training process of the models. To mitigate this threat and enhance the stability of our experimental results, we conducted a statistical analysis. Specifically, we conducted multiple runs of all experiments, repeating the training and evaluation procedures ten times. By adopting this approach, our experimental findings acquire heightened reliability and stability. Furthermore, we calculated the statistical significance of our experimental results, providing further evidence for the validity and generalizability of our results.

*Threats to External Validity.* The external threats to validity in our study primarily stem from the generalizability of our findings to other models and video datasets. To address this concern, we carefully selected a diverse set of models and video datasets for our experimental evaluation. By incorporating various model-dataset pairs, we aimed to capture a broad spectrum of scenarios and ensure that our findings are not limited to a specific combination of models and datasets. We intentionally included both natural and noisy inputs during testing. More specifically, we leveraged well-established noise generation techniques from publicly available studies. These techniques, derived from the literature on image and video processing (Shorten and Khoshgoftaar 2019; Perez and Wang 2017; Mikołajczyk and Grochowski 2018; Taylor and Nitschke 2018), enable us to augment the diversity of the video datasets used for evaluation. By incorporating these augmentation techniques, we aimed to evaluate the effectiveness of VRank on noisy contexts.

## Related Work

We present the related work in three aspects: test prioritization in DNN testing, deep neural network testing, and test prioritization for traditional software.

### Test Prioritization in DNN Testing

In the domain of DNN testing, test prioritization (Feng et al. 2020; Weiss and Tonella 2022; Dang et al. 2023, 2024; Li et al. 2024) has emerged as a critical task for identifying possibly-misclassified test inputs. Various metrics have been proposed for this purpose. DeepGini, proposed by Feng et al. (2020), aims to prioritize tests based on model uncertainty. DeepGini assumes that a test input is more likely to be mispredicted if the DNN outputs similar probabilities for each class. Byun et al. (2019) evaluated several white-box metrics for prioritizing bug-revealing inputs, including widely-used metrics like softmax confidence, Bayesian uncertainty, and input surprise. Moreover, Weiss and Tonella (2022) performed a comprehensive investigation of various DNN test input prioritization techniques, including several uncertainty-based metrics such as Vanilla Softmax, Prediction-Confidence Score (PCS), and Entropy. These metrics have been shown to be effective in identifying possibly-misclassified test inputs and aiding test prioritization efforts. Recently, Wang et al. (2021) proposed PRIMA, which uses intelligent mutation analysis for prioritizing test inputs. This approach can be applied not only to classification but also to regression models and can handle test inputs generated from adversarial input generation approaches that increase the probability of the wrong class. While PRIMA has demonstrated its effectiveness on image data, it cannot be used to prioritize video tests since its mutation rules are not adapted to video data.

The aforementioned uncertainty-based test prioritization methods can be adapted for test prioritization of video datasets. However, video data possesses unique characteristics, particularly temporal information, which necessitate a tailored test prioritization strategy. In comparison to these existing approaches, our proposed VRank introduces a carefully-designed feature generation strategy specifically for video samples. VRank leverages frame sampling techniques (Team 2023) and the ResNet model (He et al. 2016) to extract frame representations that capture the temporal information embedded within video data. By exploiting these techniques, VRank enables the effective prioritization of video tests by considering the temporal dynamics and dependencies present in the video content, thereby augmenting the accuracy and relevance of the test prioritization process.

### Deep Neural Network Testing

DNN Testing (Humbatova et al. 2021; Jahangirova and Tonella 2020) aims to systematically assess and enhance the reliability and robustness of neural network models through rigorous testing methodologies. In addition to test input prioritization, numerous approaches have been proposed to enhance the efficiency of DNN testing through the process of test selection. Test selection aims to accurately estimate the accuracy of the entire test set by labeling only a carefully chosen subset of test inputs, thereby reducing the labeling cost associated with DNN testing. By effectively selecting a representative subset of test inputs, test selection techniques can provide reliable estimates of the DNN’s performance without requiring the evaluation of the entire test set. Li et al. (2019) introduced two test selection methods, namely Cross Entropy-based Sampling (CES) and Confidence-based Stratified Sampling (CSS). CES operates by minimizing the cross-entropy between the selected set and the complete test set, thereby ensuring that the distribution of the selected test set aligns with that of the original test set. In this way, CES aims to capture the diversity and characteristics of the complete test set while using only a fraction of the available test inputs. CSS, on the other hand, leverages the confidence features of test inputs to ensure similarity between the selected test set and the entire test set. By selecting samples based on their confidence scores, CSS aims to capture the representative distribution of the test set, thereby providing accurate estimations of the DNN’s performance.

Building upon the foundation of test selection, Chen et al. (2020) proposed a practical test selection approach called Practical Accuracy Estimation (PACE). PACE integrates various techniques, including clustering, prototype selection, and adaptive random testing, to facilitate efficient and effective test selection. PACE initiates by clustering all the test inputs based on their testing capabilities. Through this process, test inputs with similar characteristics and behaviors are grouped together, enabling the identification of distinct clusters within the test set. Following clustering, prototypes are selected from each cluster using the MMD-critic algorithm (Kim et al. 2016). The MMD-critic algorithm ensures that the selected prototypes are representative of their corresponding clusters, thus capturing the diversity and variability of the test set. For test inputs that do not fall into any specific cluster, PACE employs adaptive random testing, which randomly selects samples from the remaining unclustered inputs. By adapting the sampling strategy to the unique characteristics of the unclustered inputs, adaptive random testing helps maintain the representativeness and diversity of the selected test set. It is important to note that while test selection techniques aim to reduce the labeling cost by selecting a subset of test inputs, our work primarily focuses on the complementary task of test prioritization. Unlike test selection methods that estimate the performance of a DNN by utilizing a selected subset of inputs, our proposed approach (VRank) focuses on ranking all test inputs based on their potential to reveal bugs without discarding any of them.

Besides enhancing the efficiency of DNN testing (Pei et al. 2017; Ma et al. 2018a, b; Kim et al. 2019; Ma et al. 2018c), evaluating the adequacy of DNNs has been a significant objective in several studies in the field. These studies have focused on developing metrics and frameworks to assess the coverage and effectiveness of test sets. Pei et al. (2017) introduced the concept of neuron coverage as a metric to evaluate the adequacy of a test set in covering the logic of a DNN model. Neuron coverage measures the extent to which the activations of individual neurons in the model are exercised by the test inputs. Building upon this metric, the authors developed a white-box testing framework for DNNs, which has shown effectiveness in detecting faults and revealing hidden vulnerabilities in these models.

Ma et al. (2018a) proposed DeepGauge, a comprehensive set of DNN testing coverage criteria. One of the key components of DeepGauge is neuron coverage, which serves as a significant indicator of the effectiveness of a test input. By measuring the coverage of neurons in the model, DeepGauge provides insights into the regions of the model that are adequately exercised by the test inputs. Additionally, DeepGauge introduced new coverage metrics with varying granularities to differentiate between adversarial attacks and legitimate test data. These metrics capture the subtle differences in the behavior of the model when exposed to adversarial inputs, enabling the detection and identification of such attacks. Kim et al. (2019) proposed the surprise adequacy approach for DNN testing. This approach assesses the effectiveness of a test input by quantifying its surprise with respect to the training set. The surprise of a test input is measured by the difference in the activation values of neurons in response to the new input. By evaluating the surprise of test inputs, this approach provides a means to identify inputs that exhibit unusual or unexpected behavior, highlighting potential vulnerabilities or weaknesses in the model.

### Test Prioritization for Traditional Software

Within the domain of software testing, various techniques have been explored and adopted to improve the efficiency and effectiveness of bug detection in the testing process (Lou et al. 2015; Shin et al. 2019; Papadakis et al. 2014; Tonella et al. 2006; Weiss and Tonella 2022). Among these techniques, test prioritization has gained significant attention as a means to determine the most advantageous order in which to execute test cases, aiming to detect software bugs at the earliest possible stage.

The main objective of test case prioritization is to identify the maximum number of test cases that have the potential to reveal bugs within a limited time frame. Empirical studies have demonstrated the positive impact of test case prioritization on the fault detection rate of the overall test suite (Elbaum et al. 2002; de S. Campos Junior et al. 2017; Luo et al. 2016). For instance, Di Nardo et al. (2013) conducted a case study evaluating coverage-based prioritization strategies on real-world regression faults. Their study assessed the effectiveness of various test case prioritization techniques in detecting bugs, providing insights into the efficacy of different prioritization approaches. Rothermel et al. (2001) introduced and compared three types of test case prioritization techniques for regression testing, which leveraged test execution information to determine the order of test case execution. Their research emphasized the effectiveness of these prioritization techniques in increasing the fault detection rate of the test suite. Lou et al. (2015) proposed a test case prioritization approach based on the fault detection capability of individual test cases. They introduced two models, the statistics-based model, and the probability-based model, to calculate the fault detection capability of each test case. Through their empirical evaluation, they found that the statistics-based model outperformed other approaches, highlighting the importance of considering the fault detection capability in test case prioritization.

Shin et al. (2019) developed a test case prioritization technique utilizing the diversity-aware mutation adequacy criterion. They empirically evaluated the effectiveness of mutation-based prioritization techniques using a large-scale collection of developer-written test cases. Their research shed light on the benefits of employing mutation-based prioritization techniques in practical testing scenarios. Papadakis et al. (2014) proposed a method that involved mutating Combinatorial Interaction Testing models and prioritizing test cases based on their ability to detect and eliminate mutants. They demonstrated a strong correlation between the number of model-based mutants killed and the identification of code-level faults by the test cases, illustrating the potential of model-based prioritization approaches in software fault detection.

These studies collectively showcase the effectiveness and benefits of test prioritization techniques in detecting software faults and optimizing the overall software testing process. By strategically ordering the execution of test cases, testers can allocate their limited resources more efficiently and uncover bugs earlier, leading to improved software quality and reliability.

### Prediction Techniques for Time-Series Analysis

In the literature (Ahmed et al. 2023; Van Den Oord et al. 2016; Salinas et al. 2020; Wang and Guan 2024), several notable techniques have been proposed for time-series analysis. Ahmed et al. (2023) provided a comprehensive tutorial on the use of Transformers in time-series analysis. They explained the fundamental concepts of self-attention and multi-head self-attention mechanisms, emphasizing their ability to tackle time-series tasks. Through various examples and empirical studies, they demonstrated that Transformers offer an efficient alternative to traditional methods like RNNs and LSTMs. Van Den Oord et al. (2016) introduced WaveNet, a generative DNN model that uses dilated causal convolutions to effectively capture long-range temporal dependencies. This architecture allows WaveNet to model complex temporal structures in time-series data, making it highly suitable for various time-series analysis tasks.

Salinas et al. (2020) introduced DeepAR, a probabilistic forecasting model using auto-regressive recurrent networks to handle large volumes of time series data. DeepAR can effectively capture the probabilistic nature of future values, allowing for more accurate and reliable demand forecasts. Wang and Guan (2024) proposed a novel approach for time series prediction utilizing a Multiscale Convolutional Neural-based Transformer Network. This network integrates multiscale extraction and multidimensional fusion frameworks to effectively capture multiple time-scale dependencies and the correlations among input variables. Their empirical studies demonstrated that MCTNet can significantly improve the accuracy of time series predictions, outperforming several state-of-the-art approaches on challenging datasets.

## Conclusion

To solve the labeling-cost problem specifically in the context of video test inputs, we proposed a novel test prioritization approach called VRank. The primary objective of VRank is to assign higher priority to video test inputs that are more likely to be misclassified. The fundamental concept underlying VRank is that test inputs situated closer to the decision boundary of the model are at a higher risk of being predicted incorrectly. To capture the spatial relationship between a video test and the decision boundary, we employ a vectorization technique that transforms a given video test into a lower-dimensional space to indirectly reveal the underlying proximity between the test and the decision boundary. To implement this vectorization strategy, we generate four different types of features for each video-type test: temporal features, video embedding features, prediction features, and uncertainty features. Each of these feature types captures essential aspects of the video tests and the model’s classification behavior specific to videos. Temporal features capture the unique temporal coherence inherent in a given video-type test. Video embedding features encapsulate the inherent information within the videos, while the prediction features focus on the model’s classification information regarding the videos. Uncertainty features, on the other hand, take into consideration the level of uncertainty associated with the model’s classification outputs. By combining these feature types, VRank effectively constructs a comprehensive feature vector for each individual test input. To assess the misclassification likelihood of each test input, VRank employs a LightGBM-based ranking model that takes the constructed feature vector as input and generates a misclassification score. A higher misclassification score indicates a higher probability of the test input being incorrectly predicted by the model. Based on these misclassification scores, VRank sorts all the tests within the test set in descending order, establishing a prioritized ranking. To assess the effectiveness of VRank, we carried out an empirical evaluation, comparing it with several test prioritization methods. Our evaluations involved 120 subjects, incorporating both natural and noisy data. The results of our experiments demonstrate the effectiveness of VRank in comparison to a diverse range of existing test prioritization approaches. Specifically, VRank yielded an average improvement of 5.76%$\sim$46.51% on natural datasets and 4.26%$\sim$53.56% on noisy datasets.

## Data Availability

The datasets and code used in the present study are available in our repository: https://github.com/yinghuali/VRank

## Footnotes

1 https://github.com/yinghuali/VRank
